# Expression and Potential Role of MMP-9 in Intrauterine Adhesion

**DOI:** 10.1155/2021/6676510

**Published:** 2021-01-29

**Authors:** Congqing Li, Wenyan Wang, Shiying Sun, Youjiang Xu, Ziang Fang, Lin Cong

**Affiliations:** ^1^Department of Obstetrics and Gynecology, The Second Hospital of Anhui Medical University, No. 678 Furong Road, Hefei, Anhui 230601, China; ^2^Department of Obstetrics and Gynecology, The First Affiliated Hospital of Anhui Medical University, No. 218 Jixi Road, Hefei 230022, China

## Abstract

**Objective:**

Intrauterine adhesions affect menstruation and fertility, and endometrial fibrosis is the final manifestation of IUA. MMP-9 is closely related to fibrosis. The purpose of the study was to assess the role of MMP-9 in intrauterine adhesion (IUA) in rats and patients.

**Methods:**

40 rats and 24 women were enrolled in this study. 40 rats were randomly divided into 3 groups: IUA group (*n* = 20), sham group (*n* = 10), and control group (*n* = 10). Rat IUA models were established by intrauterine mechanical and chemical injured. In this study, 12 patients of intrauterine adhesions were detected and underwent TCRA (transcervical resection of adhesion) surgery, and endometrial tissue specimens were obtained during operation. One month later, an office hysteroscopy procedure was performed, and endometrial tissue specimens were obtained during operation again (postoperative group). A group of 12 normal age-matched control individuals served as controls underwent hysteroscopy and endometrial sampling. We used immunohistochemistry to detect MMP-9 expressions in rats and human endometrial tissues and to detect MMP-9 protein levels by Western blotting. In addition, we detected mRNA expression levels with qRT-PCR.

**Results:**

The expression of MMP-9 in the IUA rats was reduced compared with that in the sham group and Ctrl group (*P* < 0.05), and the expression of MMP-9 was also reduced in the IUA patients compared with that in the Ctrl group (*P* < 0.05). The mRNA levels of MMP-9 in the endometrium reflected similar results (*P* < 0.05). The MMP-9 clearly increased even in the endometrium after TCRA surgery (*P* < 0.05).

**Conclusion:**

Our study suggests that MMP-9 may play an important role in IUA. In the future, more in-depth research should be conducted on MMP-9.

## 1. Introduction

Intrauterine adhesion (IUA) is a condition that was identified more than a century ago [1]. IUA is a very common problem encountered in clinical practice and is the main cause of menstrual volume reduction, infertility, and recurrent abortion. It has been identified that uterine cavity injury, especially induced abortion, can lead to endometrial basal layer injury. Injury is one of the direct causes of uterine cavity adhesion, and endometrial fibrosis is the final manifestation of IUA [2]. Fibrosis is a common and difficult problem to treat in the clinic. During the healing process of normal wounds, the deposition of extracellular matrix (ECM) leads to the occurrence and development of tissue fibrosis. The excessive accumulation or degradation of ECM components in the organs leads to an increase in ECM, fibrosis of the tissue, and ultimately to a decrease in or loss of function due to liver fibrosis, kidney fibrosis, pulmonary fibrosis, and intestinal fibrosis [3]. The mechanism of endometrial fibrosis due to intrauterine adhesions is still unclear. According to conventional speculation, the persistence of ECM components and the reduced deposition or degradation of the extracellular matrix may be the main causes of fibrosis [3]. The process of fibrosis involves many factors and is intricate. Fibrosis involves the deposition of ECM proteins and related molecules/factors that crosslink various ECM elements, the hydrolysis of ECM proteins, and the enzymatic degradation of ECM. Among proteolytic ECM enzymes, matrix metalloproteinases (MMPs) have been a frequent topic of research by scholars [3]. MMPs play a key role in the balance between fibrosis and antifibrosis; when fibrosis and antifibrosis are out of balance, and the degradation of the extracellular matrix eventually leads to the generation of tissue fibrosis [4, 5]. MMP-9 is a member of the metzincin family of mostly extracellular proteases. Although all of these enzymes might be promiscuous in their targeting of proteins, MMP-9 is a particular concern of researchers [6–9]. We aimed to investigate whether MMP-9 may also be involved in fibrosis in IUA. In this study, we evaluated the potential role of MMP-9 in fibrosis in IUA by measuring the expression of MMP-9 in endometrial tissues.

## 2. Materials and Methods

**Figure 1:**
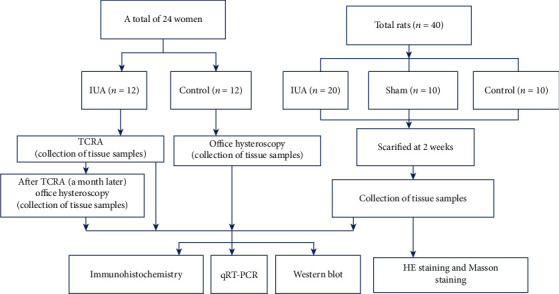
Master plan route for this study.

The master plan for this study is shown in Figure 1.

### 2.1. IUA Patients and Controls

24 patients were included in our study, of which 12 were IUA patients. The ethics committee of the Second Hospital of Anhui Medical University approved the research plan (PJ-YX2019-016F1). The average age of the IUA patients was 29.5 years (24-40 years), and that of the control group was 24.75 years (21-39 years). We collected samples of the endometrial fibrosis tissue from May 2018 to July 2018. IUA patients were scored and graded according to criteria designed by the American Fertility Association (AFS) [10]. 12 patients underwent TCRA (transcervical resection of adhesion) surgery, and endometrial tissue specimens were obtained during operation (IUA group, *n* = 12). One month later, an office hysteroscopy procedure was performed, and it can be used to evaluate the uterine cavity and obtained endometrial tissue specimens (TCRA postoperative group, *n* = 12). A group of 12 normal age-matched control individuals served as controls underwent hysteroscopy and endometrial sampling. The criteria for the inclusion of surgical specimens excluded the presence of infection, any disease of the uterus, chronic inflammation, and malignant diseases. All patients signed written informed consent forms before surgery and agreed to the use of endometrial tissue specimens for scientific research.

### 2.2. Rat Experimental Protocol

The experimental animals used for this project were purchased from the Animal Experimental Center of Anhui Medical University under the animal certificate number: SCXK (Anhui) 2005-001. The experimental protocol followed the requirements of animal ethics and was implemented in accordance with the regulations of the Animal Ethics Committee of Anhui Medical University. The studies were approved by the Institutional Animal Care and Use Committee at the Anhui Medical University (LLSC20180085). From January 5th to February 26th, 2017, 40 mature fertile female SD rats were enrolled in this study (weighing 200–250 g, age 8 weeks). The experimental animals were kept in the animal breeding room of the Experimental Center of the Second Affiliated Hospital of Anhui Medical University. Under standard laboratory conditions, the ambient temperature of the animal breeding room is 25 ± 2°C, and the maximum temperature difference must not exceed 2°C; the humidity is controlled at 60% ±, the indoor noise is less than 55 decibels (dB), and the rats were given one week to adapt to the breeding environment.

#### 2.2.1. IUA Rat Models

The experimental surgery in all rats was performed by the same person, and the method of anesthesia was intraperitoneal administration of sterile sodium pentobarbital (30 mg/kg). Our goal was to use the rat uterus to generate an animal model to simulate human uterine adhesions. In clinical work, phenol mucus is used in female sterilization surgery and is safe and reliable [11]. 40 rats were randomly divided into three groups. Rats were sacrificed 2 weeks after surgery via the administration of carbon dioxide. Then, the uterus was removed, and the endometrial tissue specimens were obtained. After the specimens were removed, the bodies of the rats were harmlessly treated.

#### 2.2.2. IUA Model Group, Sham Group, and Control Group

**Figure 2:**
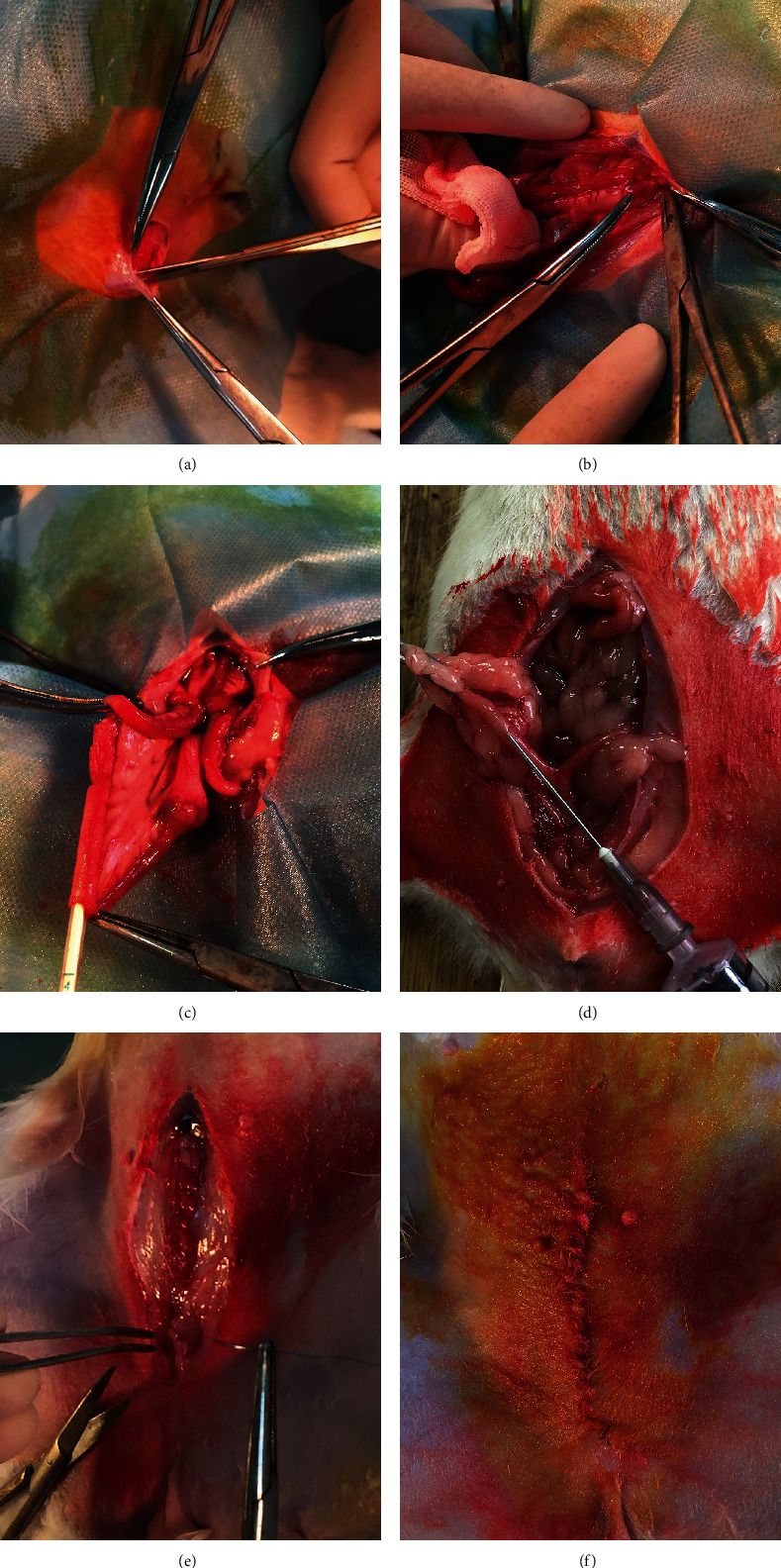
The operation procedure of the IUA rat model. (a) Select the incision sign. (b) Incise the skin and subcutaneous tissue. (c) Rat uterus was observed, and one side uterine cavity was probed. (d) Phenol mucilage was slowly injected into rat uterine cavity with syringe. (e) After the operation, the abdominal incision was closed. (f) After suturing the skin, sterilize the incision again.

20 rats were included in the IUA group. We used a uterine cavity from one rat as the experimental research object and injected 0.04 ml of phenol mucilage into the selected uterine cavity; the phenol mucilage was composed of 25% v/v phenol solution, 5% v/v gum Arabic, and 20% glycerol v/v. This method resulted in the formation of adhesions within 2 weeks. The surgical procedure is shown in Figure 2. 10 rats were subjected to sham surgery, and the surgical procedure was the same as that used for the IUA group; the only difference was that normal saline was injected into the right uterine cavity instead of phenol mucilage. The control group also comprised 10 rats, which were not injected in the right uterine cavity.

### 2.3. Collection of Tissue Samples from Patients and Rats

The endometrial tissue samples were divided into two groups: one group was kept at room temperature in formalin, and the other was stored at -80°C until use.

### 2.4. HE Staining and Masson Trichrome Staining of the Rat Tissue Samples

The tissue specimens were fixed in 4% neutral buffered formalin, and paraffin sections were routinely made and then stained according to the HE staining procedure. The Masson trichrome staining methods and procedures were performed according to the instructions of the reagent manufacturer [11].

### 2.5. Immunohistochemistry of Human and Rat Tissue Samples

Tissue specimen sections were stained with conventional immunohistochemistry (IHC) procedures [12, 13], and the IHC reagents were used according to the manufacturer's instructions (ZhongShanJinQiao, Beijing, China). To measure the relative expression of MMP-9 in different groups of endometrial tissues, we calculated the expression score by evaluating the percentage of positive cells and the intensity of the staining signal. The expression score was calculated by multiplying the percentage of positive cells by the intensity score and then converting the result to determine the relative expression.

### 2.6. Western Blot Analysis

Western blotting was performed according to the manufacturer's instructions (Beyotime, Shanghai, China). Molecular imaging systems (Bio-Rad, Philadelphia, PA, USA) were used to visualize the bands, and finally, the relative expression value was calculated. Repeat three times for each sample.

### 2.7. Analysis of MMP-9 mRNA by RT-qPCR

Total RNA was extracted using TRIzol reagent (Invitrogen), and an RT kit (Takara) was used for the reverse transcription reactions according to the instructions. The MMP-9 mRNA was detected by PCR using cDNA as a template. Real-time quantitative PCR was used to detect the relative mRNA levels. The internal control was GAPDH. The primer sequences are shown in Table 1. The PCR conditions consisted of 5 min at 95°C for one cycle followed by 45 cycles of 95°C for 10 s, 60°C for 40 s, and 72°C for 90 s. Repeat three times for each sample.

### 2.8. Statistical Analyses

All statistical analyses were performed using SPSS software (version 19.0, SPSS, Chicago, IL), and the differences between groups were analyzed using Student's *t*-test, the Mann–Whitney *U* test, or one-way analysis of variance with the Kruskal-Wallis test for correction. A *P* value <0.05 was considered to be statistically significant.

## 3. Results

### 3.1. Clinical Characteristics of Patients with IUA

12 patients with intrauterine adhesions were included in this study, and their average age was 29.5 years. The IUA grade was 83% severe (score ≥9 points) and 17% moderate (score 5-8 points) according to the American Fertility Association (AFS) criteria. 6 of the 12 patients with intrauterine adhesions had children, but one of them was stillborn, and they all have fertility needs. TCRA was performed under general anesthesia. One month later, outpatient hysteroscopy was performed to evaluate the uterine cavity morphology. 8 cases had filmy membranous adhesions at the bottom of the uterus, and 4 cases had dense uterine segment adhesion. During the hysteroscopy procedure, the adhesion tissue is bluntly separated through the sheath. Patients' clinical data are shown in Table 2.

### 3.2. HE Staining and Masson Trichrome Staining of Rat Tissue Samples

**Figure 3:**
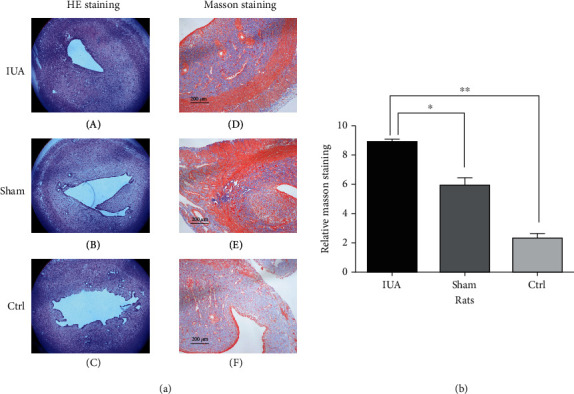
(a) HE and Masson staining revealed in rats. (A, D) IUA group (phenol mucilage treatment), (B, E) sham group (saline treatment), and (C, F) control group (no treatment), scale bar = 200 *μ*m. (b) Relative Masson staining in rats, comparison of IUA to sham and Ctrl, ^∗^*P* < 0.05, ^∗∗^*P* < 0.05.

HE staining showed a narrowing of the uterine cavity in the IUA group. The Masson trichrome staining results showed that more severe fibrosis occurred in the endometrial tissue of the IUA group compared with that of the control group and the sham operation group (Figure 3).

### 3.3. MMP-9 Expression in IUA Rats and Patients

**Figure 4:**
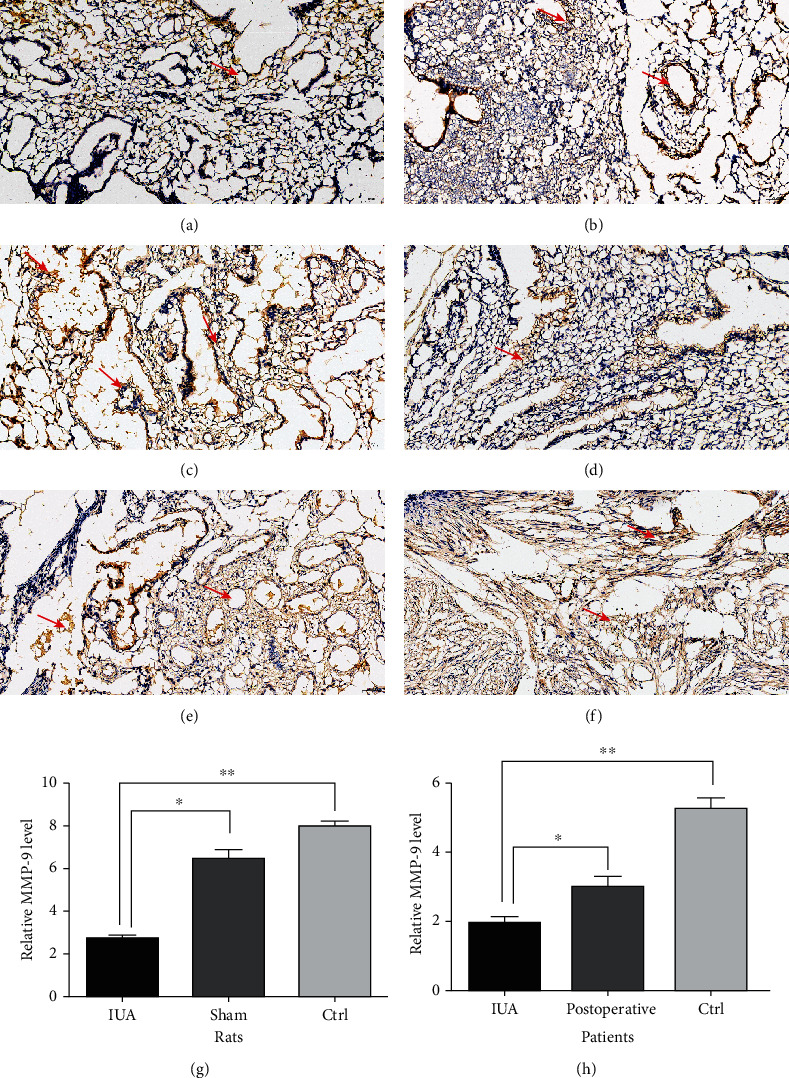
IHC of MMP-9 in rats and patients. Rat groups: (a) (IUA), (b) (sham), and (c) (control). Patient group: (d) (IUA), (e) (postoperative), and (f) (control). Scale bar = 50 *μ*M. (g) Relative MMP-9 level in rats, comparison of IUA to Sham and Ctrl, ^∗^*P* < 0.05, ^∗∗^*P* < 0.05. (h) Relative MMP-9 level in patients, comparison of IUA to postoperative and Ctrl, ^∗^*P* < 0.05, ^∗∗^*P* < 0.05.

**Figure 5:**
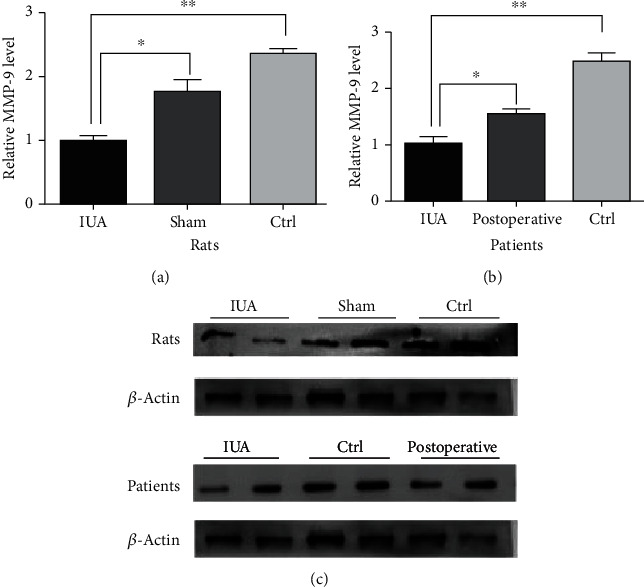
Determination of the MMP-9 protein expression in IUA rats and patients. (a) Relative MMP-9 protein expression in rat groups. Comparison of IUA to Ctrl and sham group, ^∗^*P* < 0.05, ^∗∗^*P* < 0.05. (b) Relative MMP-9 protein expression in patients. Comparison of IUA to postoperative and Ctrl, ^∗^*P* < 0.05, ^∗∗^*P* < 0.05. (c) Gel electrophoresis picture of MMP-9.

**Figure 6:**
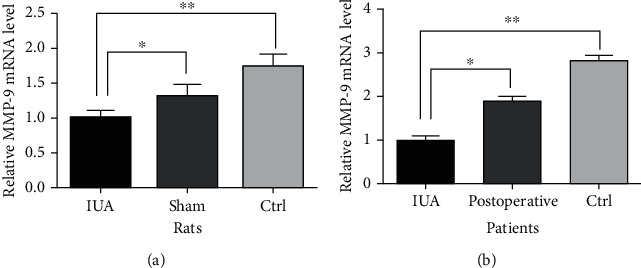
qRT-PCR analysis of the MMP-9 mRNA expression. (a) IUA rats. ^∗^*P* < 0.05, ^∗∗^*P* < 0.05. (b) IUA patients, comparison of IUA to postoperative and Ctrl, ^∗^*P* < 0.05, ^∗∗^*P* < 0.05.

It is known that MMP-9 plays an important role in fibrosis, and we measured the tissue sample expression by IHC, protein detection, and RT-PCR of MMP-9 mRNA in IUA rats and patients to assess whether there was differential expression of MMP-9 in IUA. We performed IHC staining of the tissue samples from IUA rats and patients, which revealed that the IUA groups were negative for MMP-9 staining. The differences were significant in the analysis (*P* < 0.05) (Figure 4). We detected the protein expression of MMP-9 by Western blotting and observed significant decreases in the protein expression in IUA rats and patients compared to that in controls. The MMP-9 protein expression was significantly different (*P* < 0.05) (Figure 5). In addition, the mRNA expression of MMP-9 was significantly decreased (*P* < 0.05) in IUA rats and patients compared with that in controls (*P* < 0.05) (Figure 6).

## 4. Discussion

In general, massive granulation, tissue hyperplasia, and fibrosis in the uterine cavity after abortion or curettage and IUAs occur 5 to 7 days after injury. When a sufficient amount of fibrosis occurs, regulatory mechanisms can hinder the regeneration of the endometrium and cause the formation of intrauterine adhesions [14]. Generally, in the initial stage of tissue damage, damaged and dead cells release antifibrinolytic coagulation factors, which trigger platelet activation, generate high levels of MMPs, destroy the ECM, and allow inflammatory mediators to recruit inflammatory cells to the injury site. On the other hand, the microenvironment of the injury site will also change accordingly. The proinflammatory response will lead to the activation of matrix-producing cells and will also enhance the formation of fibers [4]. The MMP-9 expression is not isolated in the body; it is affected by other members of the MMP family, and it is controlled by metalloproteinase tissue inhibitors (TIMPs) [4]. MMP-9 has been extensively studied by scholars, especially in the fibrosis of the lung, liver, and heart [15]. Basic research has found that MMPs are not only a physiological effector of extracellular matrix turnover but also a key factor in the remodeling process under pathological conditions [16, 17]. The specific details of the fibrosis process are unknown, but we should realize that the functions of specific MMPs may not be the same in different organ systems [18].

IUA is the manifestation of tissue fibrosis. Here, we found that the MMP-9 expression was decreased in both IUA rats and patients. IHC, Western blotting, and RT-qPCR were used to detect MMP-9 mRNA and protein. Saribas and colleagues used a needle tube to injure the uterine cavity to establish a rat IUA model. Twenty-four rats were divided into four groups on average. The endometrium was obtained from the IUA group for the detection of MMP-9 and other related tests 8 weeks after modeling. Based on the IUA model, stem cells and exosomes were used for follow-up intervention. MMP-2 and MMP-9 expression were enhanced by MSC and exosomal therapy; in addition, the TIMP-2 expression was decreased [19]. Despite the different damage formation methods, the end results were quite similar. Of course, this study was simply based on the IUA rat model, and the time point for obtaining the endometrium was fixed. For human IUA patients, the expression of MMP-9 and TIMPs in the endometrium may not be consistent with those in animal models due to the differences in the IUA time course. In our study, the phenol mucilage method was used to establish the IUA model of rats, and MMP-9 detection was performed, the results of which were consistent with the previous research results. At the same time, we also collected specimens from patients with different disease durations for MMP-9 detection in the endometrium. The study results did not change significantly due to the length of the disease course. Human IUA endometrium showed low expression of MMP-9. Coincidentally, Chen and colleagues also used 24 rats to study uterine adhesions. The rats were divided into 2 groups on average. For the IUA group, the endometria of the left uteri were scraped without treatment. The right uteri were used as the control group. At 3, 7, 14, and 28 days after transplantation, the uteri were sampled for evaluation. Postoperative test results showed that the expression of MMP-9 in the endometrium was increased, but the thickness of endometrial glands and endometrium in fibrotic tissues was not different from those in the IUA group [20]. Our study found that MMP-9 showed low expression in the endometrial tissue of IUA rats, which is consistent with the previous two results [19, 20]. The value of freeze-drying the amniotic membrane was assessed using the rat IUA model. The expression of MMP-9 in human IUA intima was not tested. In the rat models and IUA patients, why was MMP-9 expressed at a low level? The reasons may be as follows. First, the MMP-9 expression changes dynamically in different stages of fibrosis and may be high in the early stage. MMP-9 appears to be downregulated as fibrosis progresses. However, the rat animal model did not show that the MMP-9 expression changes significantly with the operation time [20]. Second, in this study, perhaps the degree of fibrosis in IUA had an impact on the results, and the IUA patients included in this study all had third degree fibrosis, with certain limitations. In addition, patients with intrauterine adhesions have an urgent desire for childbirth. The classical therapeutic approaches for infertile patients with IUA are hysteroscopic adhesiolysis, removing visible intrauterine adhesions [21]. In this study, the uterine cavity after intrauterine adhesion separation was significantly improved. MMP-9 in the endometrium after operation also increased, and the difference is statistically significant. It further suggests that MMP-9 is involved in the occurrence of IUA. Further study is needed to confirm the involvement of the intracellular MMP-9 signaling pathway in IUA, especially to identify and verify other key factors and the relationship between these key factors, such as MMP-2, MMP-3, MMP-7, IMP-1, and TIMP-2.

## 5. Conclusion

In summary, this study established an animal model for studying IUA mechanisms. In addition, we determined that MMP-9 is an important factor involved in IUA. In future research, we can carry out follow-up studies on MMP-9 to explore new ideas for mechanisms in IUA.

## Figures and Tables

**Table 1:** Primers used for PCR analysis.

Target mRNA	Primer sequence
MMP-9-forwards: MMP-9-reverse:	5′-TTGACAGCGACAAGAAGTGG-3′ 5′-CCCTCAGTGAAGCGGTACAT-3′
GADPH-forwards: GADPH-reverse:	5′-GGTTGAGCAGGTACTTT-3′ 5′-AGCAAGAGCACAAGAGGAAG-3′

**Table 2:** IUA patients' clinical data.

IUA patient	Age	Pregnancy history	Before TCRA surgery		After TCRA surgery	
			Grade	Score	Grade	Score
1	34	Gravida 1, Para 0	Severe	9	Mild	2
2	28	Gravida 4, Para 1	Severe	10	Moderate	6
3	35	Gravida 2, Para 1	Severe	10	Mild	1
4	32	Gravida 2, Para 0	Moderate	8	Mild	4
5	25	Gravida 1, Para 1 (a stillbirth,2016, fetal weight 5 kg)	Severe	10	Mild	3
6	34	Gravida 3, Para 1	Severe	10	Mild	2
7	30	Gravida 2, Para 0	Severe	9	Moderate	5
8	24	Gravida 1, Para 0	Severe	10	Mild	3
9	40	Gravida 3, Para 1	Severe	10	Mild	2
10	28	Gravida 2, Para 0	Severe	9	Moderate	5
11	29	Gravida 4, Para 0	Moderate	8	Mild	2
12	35	Gravida 6, Para 1	Severe	9	Moderate	5

## Data Availability

Data supporting these study findings are available from the corresponding authors upon reasonable request.

## Abbreviations

IUA: Intrauterine adhesion
MMP-9: Matrix metalloproteinases 9
IHC: Immunohistochemistry
mRNA: Messenger RNA.
